# Current Advances in Diagnostic and Treatment Approaches for Subclavian Steal Syndrome: A Case Report and Review of the Literature

**DOI:** 10.7759/cureus.65925

**Published:** 2024-08-01

**Authors:** Sudhanshu Tonpe, Himandri Warbhe, Pankaj Banode, Nikhita Gaddam

**Affiliations:** 1 Department of Interventional Radiology, Jawaharlal Nehru Medical College, Datta Meghe Institute of Higher Education and Research, Wardha, IND; 2 Department of Respiratory Medicine, Jawaharlal Nehru Medical College, Datta Meghe Institute of Higher Education and Research, Wardha, IND; 3 Department of Radiology, Government Medical College, Nirmal, Nirmal, IND

**Keywords:** angioplasty, stent, subclavian artery, case report, subclavian steal syndrome

## Abstract

With newly created therapy devices and cutting-edge diagnostic techniques, we successfully diagnosed and treated subclavian steal syndrome in this case report. This case report is complemented by a literature review that examines the current state of knowledge about diagnostic and treatment options. The patient reported pain and numbness in his left upper arm when raising his arm above his head. On clinical examination, he had good left radial and ulnar pulses while in a sitting position; however, he had absent left ulnar pulses when he raised his hand above his head. Angiography revealed retrograde perfusion of the left vertebral artery and nearly complete occlusion of the ostium of the left subclavian artery. The patient underwent angioplasty and stenting. Immediately after the procedure, the patient reported a reduction in the pain and numbness in his left upper limb by 50%, which completely disappeared at his routine follow-up after one month. The patient was completely asymptomatic during follow-up and had no signs of neurological deficit.

## Introduction

Subclavian steal syndrome (SSS) manifests as flow reversal within a branch of the subclavian artery, triggered by a hemodynamically significant obstruction or severe constriction in the proximal subclavian artery. Contorni first documented it, followed by Reivich et al. [1,2]. This condition, which Fisher refers to as SSS, involves the diversion of blood from the vertebral artery through a process commonly referred to as "stealing," where blood is redirected from the contralateral vertebral artery to the opposite vertebral artery [3]. On the other hand, subclavian stenosis is typically asymptomatic and does not call for special treatment aside from that which addresses the underlying cause [4,5]. Some individuals affected by SSS might manifest indications of cerebral arterial insufficiency, typically presenting as transient cerebral ischemia [5]. The diagnosis of SSS requires a precise assessment of the discrepancies in blood pressure (BP) across both arms and the retrograde circulation in the vertebral artery [6,7]. Last but not least, SSS is confirmed by aortography. Subclavian artery stenosis was previously treated with balloon catheter percutaneous angioplasty [8,9]. Currently, stent insertion is usually utilized to treat subclavian stenosis due to the limitations of angioplasty alone [10]. With newly created therapy devices and cutting-edge diagnostic techniques, we successfully diagnose and treat SSS in this case report. Additionally, a brief overview of SSS and the most recent developments in its diagnosis and management is provided.

## Case presentation

A male patient, aged 40, was hospitalized for a planned surgical procedure to remove a cataract in his right eye. The patient also reported pain and numbness in his left upper arm when raising his arm above his head during his pre-anaesthesia examination. On clinical examination, he had good left radial and ulnar pulses while in a sitting position; however, he had absent left ulnar pulses when he raised his hand above his head. He had no history of syncope or dizziness. The patient's heart rate was measured at 76 beats per minute. His BP in his right arm and left hand was measured at 120/74 mmHg and 88/60 mmHg, respectively. His neurological and cardiac examinations, including a 12-lead electrocardiogram (ECG), respiratory examination, and lab tests, were normal. On echocardiography, the examination revealed intact full left ventricular systolic function, minor left ventricular enlargement, and reduced contractility of the interventricular septum and left ventricular apex. Brain magnetic resonance imaging (MRI) did not show any abnormal findings.

It was suggested that he should undergo a colour Doppler examination as a result of the positional absence of pulses in the left ulnar region, and further assessment was subsequently performed. Triplex ultrasonography detected a lack of blood circulation in the left ulnar artery after raising the left arm above the head. In contrast, adequate blood flow was detected in both ulnar arteries in the supine position. Flow reversal in the left vertebral artery was observed regardless of position.

**Figure 1:**
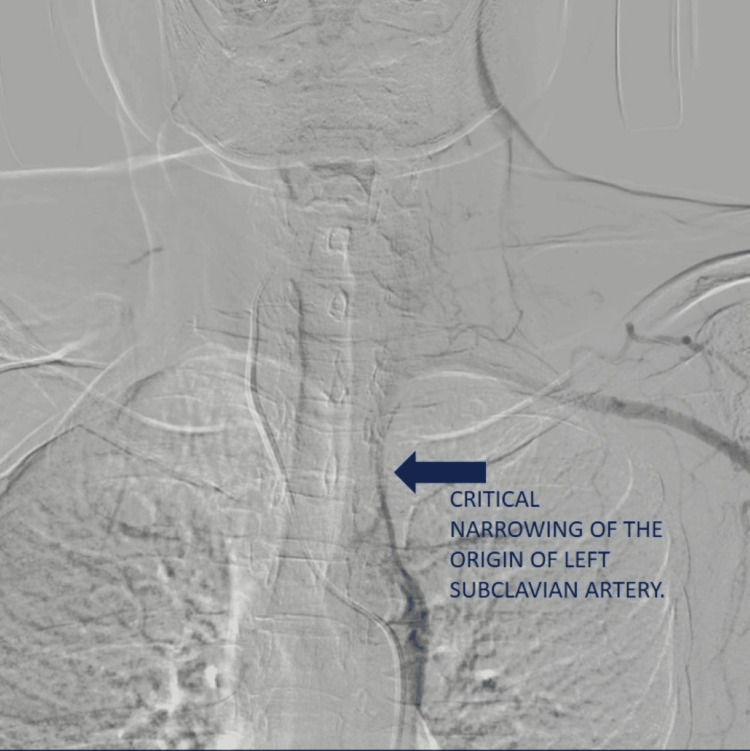
Image from the angiography showing near total occlusion of the left subclavian artery (blue arrow).

An angiography of the right vertebral artery was performed with a size 5F French-Size Head Hunter catheter (Video 1), the observation revealed retrograde perfusion of the left vertebral artery and nearly complete occlusion of the ostium of the left subclavian artery (Figure 1).

**Video 1:** Angiography of the right vertebral artery (red arrow) demonstrating retrograde filling of the left vertebral artery (orange arrow) and critical narrowing of the left subclavian artery (blue arrow).

**Figure 2:**
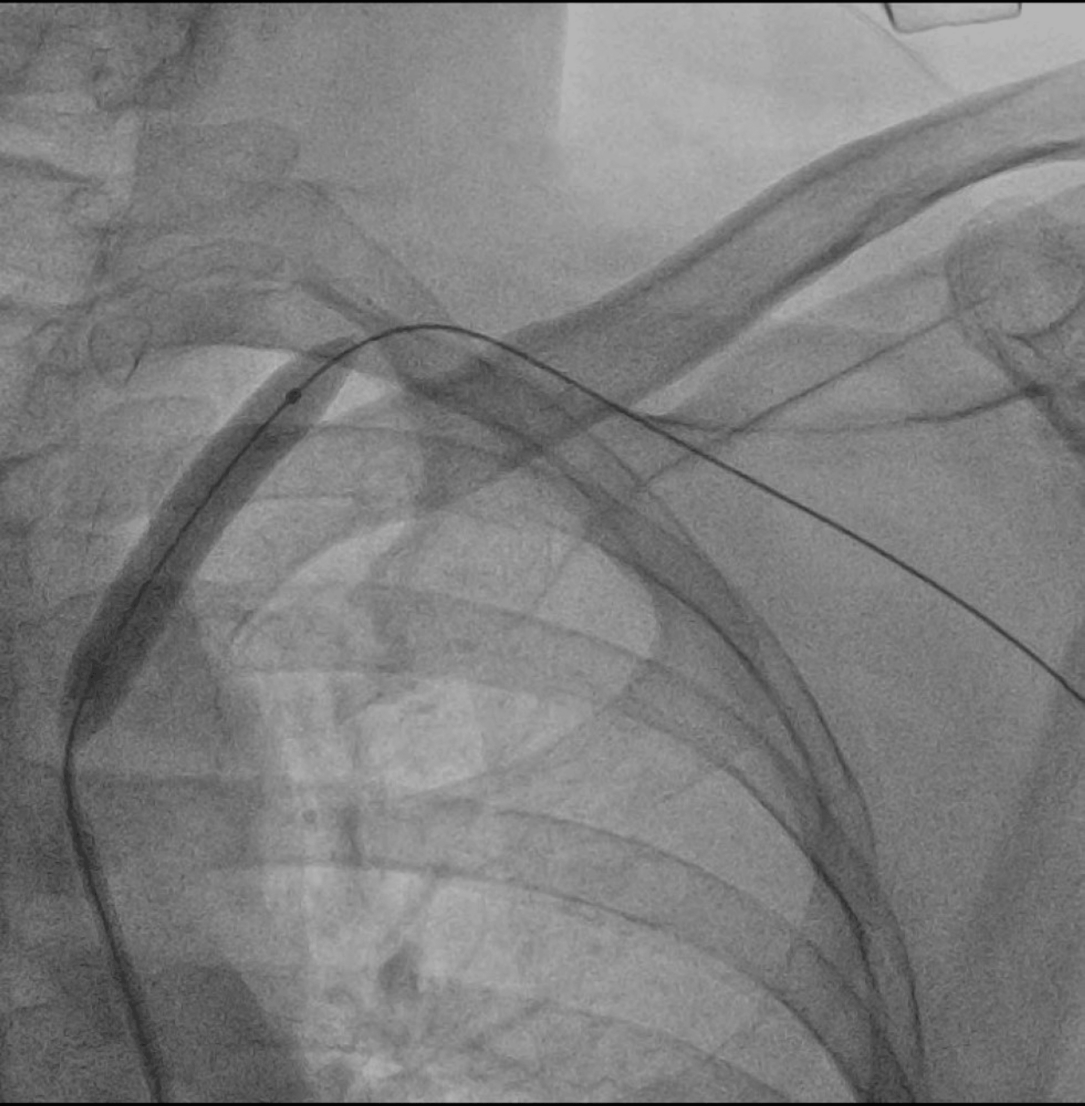
Angioplasty of the narrowed segment performed with a 5 mm x 600 mm balloon.

The radiograph of the neck and chest did not show any significant findings. Subsequently, the patient received angioplasty and stent placement in the left subclavian artery. A long 7 French Shuttle sheath was placed at the origin of the left subclavian artery, and the narrowed segment was crossed with a 0.35 hydrophilic wire. Balloon plasty of the narrowed segment was performed with a 5 mm x 600 mm balloon (Figure 2).

**Figure 3:**
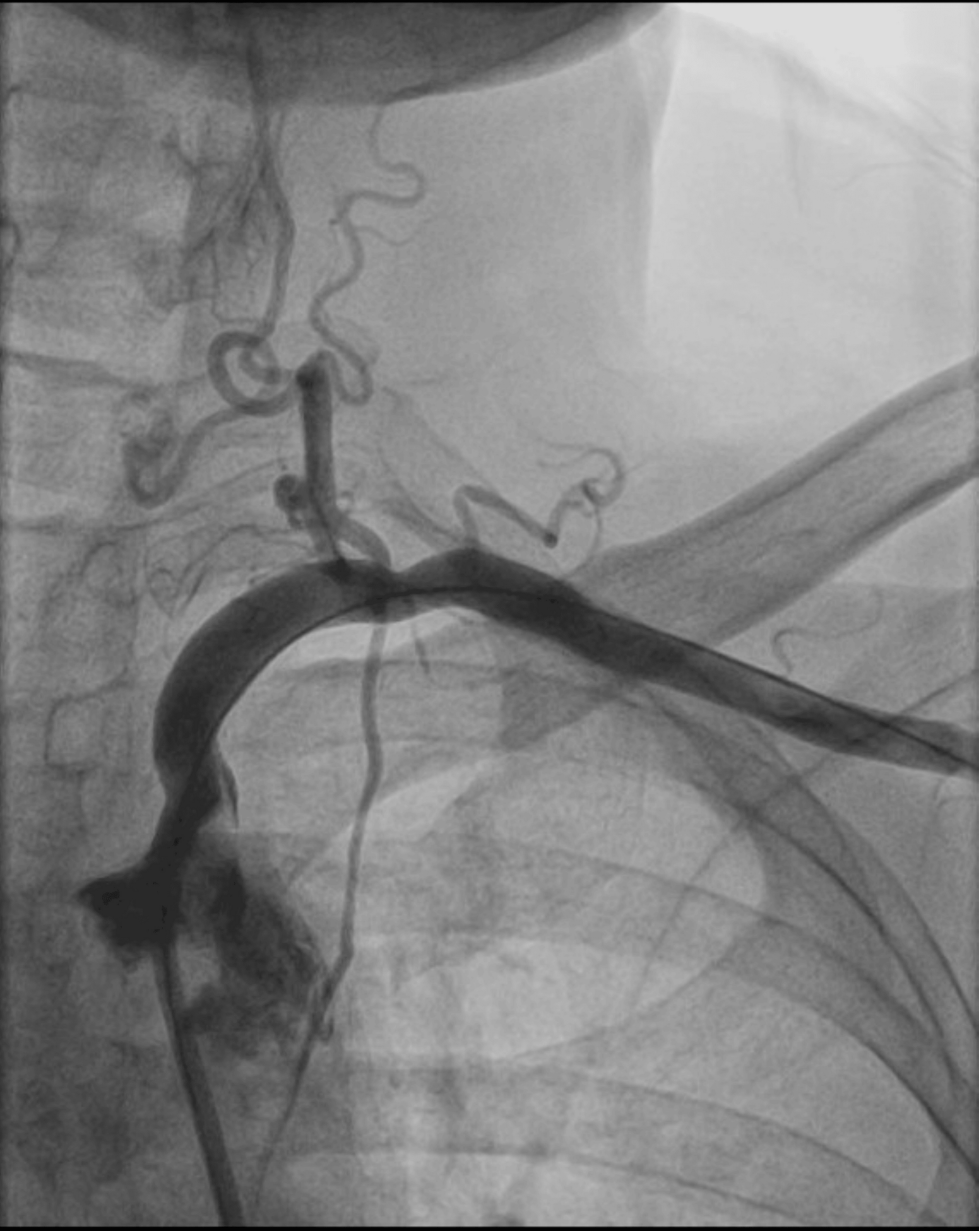
Post-angioplasty, there is flow across the narrowed segment of the left subclavian artery; however, the lumen was still critically narrowed.

Post-angioplasty angiogram demonstrated flow across the narrowed segment; however, the lumen was still critically narrowed (Figure 3).

The decision was made to place a balloon-expandable stent across the narrowing for precise placement of the stent. The stent used was a 6 mm x 39 mm Omnilink Elite™, inflated with a pressure of 6 atm using an indeflator device (Video 2).

**Video 2:** Balloon expandable stent being deployed across the narrowed segment of the left subclavian artery (red arrow).

Post-stenting, the angiogram demonstrated good flow across the narrowing (Video 3).

**Video 3:** Post-stenting, the angiogram demonstrated good flow across the narrowed left subclavian artery (red arrows).

Immediately after the procedure, the patient reported a reduction in the pain and numbness in his left upper limb by 50%, which completely disappeared at his routine follow-up after one month. The patient also underwent cataract surgery two days after stent placement, which was his primary concern. He was neurologically normal after the procedure and continued with follow-up visits.

## Discussion

We presented a case involving the fortuitous detection of SSS in conjunction with arm elevation, leading to paraesthesia in the left upper limb during occupational activities. The diagnosis of the patient was established through state-of-the-art diagnostic modalities and subsequently managed via endovascular intervention. SSS is characterized by the following criteria: (I) reversal of blood flow in the vertebral artery; (II) presence of blockage or considerable narrowing in the subclavian or innominate artery; and (III) maintenance of unobstructed blood flow in both the basilar and vertebral arteries [5]. The case we observed met all the specified criteria.

In an investigation involving 1,114 individuals who underwent arteriography and were found to have blockages in the subclavian or innominate artery, it was observed that merely 168 cases (15%) satisfied the criteria established earlier for SSS. Ninety-five per cent (159 patients) of the patients who fit the criteria exhibited symptoms, and 30 patients (18%) developed syncope [5]. Vertigo was the most common symptom, whereas syncope occurred less frequently. In a separate investigation, 45 of 500 patients exhibiting asymptomatic neck bruits yielded a positive result on the subclavian steal test, indicating that 64% of the population displayed the ailment. In comparison, 45 of the 500 patients presented with severe subclavian stenosis [4]. At the follow-up, no patient had a stroke or any symptoms associated with arm exercise during the steal test. SSS is not commonly seen in patients with subclavian stenosis. In patients with symptomatic subclavian stenosis, vertigo, dizziness, and syncope have been observed variably [5,11-15]. On the other hand, among syncope patients who underwent examination, a significant proportion (30-50%) had no aetiology identified [16-18]. Cerebrovascular disease is a rare cause of syncope, accounting for about 1% to 2% of cases. Syncope caused by SSS has been documented in 0.98% of instances (2/204 patients). Syncope secondary to vertebrobasilar transient ischemic episodes was only diagnosed when simultaneous symptoms of vertebrobasilar ischemia with momentary unconsciousness occurred [16].

Thus, in addition to syncope, the additional symptoms likely resulting from cerebral circulation ischemia were crucial in the diagnosis of SSS. Moreover, indications of upper limb manifestations, such as weakened arm function while working, indicated the presence of SSS.

A pressure difference of 10-15 mm Hg between both limbs is associated with peripheral vascular disorders and a significant mortality risk [4,19]. It is advisable to simultaneously evaluate BP in both arms to diagnose and assess SSS. In order to assess the BP difference in both arms concurrently, a volume-plethysmographic pressure and pulse wave analytical device is employed.

Demonstrating retrograde flow in the carotid and vertebral arteries is imperative when diagnosing SSS [6,20-22]. Prior to the development of Doppler ultrasound, the only way to assess cerebral artery flow was through the highly invasive procedure known as contrast medium arteriography [23]. In our case, the retrograde vertebral flow was demonstrated by the colour Doppler.

Approximately 85% of patients who underwent subclavian artery intervention utilized the right femoral approach [24]. Despite the distance between the femoral access site and the subclavian lesion, stent implantation techniques via the femoral artery have improved thanks to the introduction of novel, cutting-edge technologies like the stent, the vascular sheath, the guiding catheter, and the guidewire. Due to the radial artery's smaller diameter than that of the femoral artery, vascular damage and occlusions are significant concerns with a radial approach, despite the more superficial catheter and guidewire insertion. Consequently, a smaller guiding catheter and sheath are required. Patients who cannot have transfemoral access may benefit from transradial access, which employs a smaller vascular incision and a stent with a lower profile.

Iatrogenic problems such as guidewire entrapment, dissection, and vasospasm may arise from the use of a distal protection device [25]. As early as 1980, Bachman and Kim observed that angioplasty for subclavian artery stenosis was associated with a significant delay in restoring antegrade vertebral flow in individuals with SSS [26]. This finding was corroborated by a study that used continuous ultrasonography Doppler equipment to evaluate the ipsilateral vertebral artery flow before, during, and following angioplasty [9]. These findings indicated that restoring antegrade flow in the vertebral artery takes about 30 minutes, even when subclavian stenosis has been adequately revascularized. Delays prevent complications from vertebral thromboembolism by allowing emboli to enter the upper extremities instead of the cerebral circulation. Only those with preoperative retrograde flow in the vertebral artery exhibit this protective effect.

In our case, the antegrade vertebral flow was observed on angiography after half an hour of stenting. SSS is associated with very tight stenosis; hence, predilation angioplasty is recommended prior to stenting. Direct stenting can be done in less severe stenosis.

A randomized trial has been performed in the femoral arteries; however, no such trial has been conducted in the subclavian arteries [27]. Unlike coronary artery stents, peripheral arteries have larger diameters, and revascularization is more visible in smaller stents than in larger drug-eluting peripheral stents. In our case, the sizing of the bare-metal stent was based on the size of the subclavian artery's landing site. Further research is needed, as there currently needs to be more data regarding these comparisons in subclavian artery intervention.

Balloon-expandable and self-expanding stents have been used successfully for revascularizing blockages or significant stenosis in the subclavian artery [24]. The features of the lesion determine which kind of stent is best. Balloon-expandable stents are appropriate for lesions with a large reference vessel diameter, severe stenosis, or stiff occlusion. In the present case, we used post-stent plasty followed by the use of a balloon-expandable stent.

## Conclusions

The patient in question had an incidental presentation of syncope and left upper arm paralysis due to SSS. The patient underwent endovascular balloon-mounted stent therapy after confirming a colour Doppler and angiography diagnosis. The patient was completely asymptomatic during follow-up and had no signs of neurological deficit. The patient also underwent cataract surgery, which was his primary complaint. This review looked at the current state of knowledge about diagnostic and treatment options.
